# Patient and family financial burden associated with cancer treatment in Canada: a national study

**DOI:** 10.1007/s00520-020-05907-x

**Published:** 2021-01-05

**Authors:** Christopher J. Longo, Margaret I. Fitch, Jonathan M. Loree, Linda E. Carlson, Donna Turner, Winson Y. Cheung, Darin Gopaul, Janet Ellis, Jolie Ringash, Maria Mathews, Jim Wright, Christiaan Stevens, David D’Souza, Robin Urquhart, Tuhin Maity, Fanor Balderrama, Evette Haddad

**Affiliations:** 1 DeGroote School of Business–Health Policy & Management, McMaster University, 4350 South Service Rd, Burlington, Ontario L7L 5R8 Canada; 2 Dalla Lana School of Public Health, University of Toronto, Toronto, Canada; 3 Bloomberg Faculty of Nursing, University of Toronto, Toronto, Ontario M4C 4V9 Canada; 4 Department of Medicine, Division of Medical Oncology, BC Cancer / University of British Columbia, 600 West 10th Avenue, Vancouver, British Columbia V5Z4E6 Canada; 5 Department of Oncology, Cummings School of Medicine, University of Calgary, 2202 2nd St SW, Calgary, Alberta T2S 3C1 Canada; 6 Department of Community Health Sciences, Rady Faculty of Health Sciences, University of Manitoba, 675 McDermot Avenue, Winnipeg, MB R3E 0V9 Canada; 7 Department of Oncology, University of Calgary, 1331–29 Street NW, Calgary, Alberta T2N 4N2 Canada; 8 Grand River Regional Cancer Centre, Kitchener, Ontario N2G 1G3 Canada; 9 Odette Cancer Centre, Sunnybrook Health Sciences Centre, Toronto, Ontario Canada; 10 Princess Margaret Cancer Centre/UHN, 610 University Ave, Toronto, Ontario M5G 2M9 Canada; 11 Department of Family Medicine, Western University, London, Ontario Canada; 12 Juravinski Cancer Centre, McMaster University, Hamilton, Canada; 13 Department of Radiation Oncology, University of Toronto, Barrie, Ontario L4M 6M2 Canada; 14 London Health Sciences Centre, Western University, London, Ontario Canada; 15 Department of Surgery, Dalhousie University, Room 8-032, Centennial Building, 1276 South Park St., Halifax, Nova Scotia B3H 2Y9 Canada; 16 Department of Health Research Methods, Evidence, and Impact, McMaster University, 1280 Main Street West, Hamilton, Ontario L8S 4L8 Canada

**Keywords:** Cancer, Self-administered questionnaire, Out-of-pocket costs, Financial burden, Financial toxicity

## Abstract

**Goal:**

To determine patient-reported financial and family burden associated with treatment of cancer in the previous 28 days across Canada.

**Methods:**

A self-administered questionnaire (P-SAFE v7.2.4) was completed by 901 patients with cancer from twenty cancer centres nationally (344 breast, 183 colorectal, 158 lung, 216 prostate) measuring direct and indirect costs related to cancer treatment and foregone care. Monthly self-reported out-of-pocket-costs (OOPCs) included drugs, homecare, homemaking, complementary/ alternative medicines, vitamins/supplements, family care, accommodations, devices, and “other” costs. Travel and parking costs were captured separately. Patients indicated if OOPC, travel, parking, and lost income were a financial burden.

**Results:**

Mean 28-day OOPCs were CA$518 (US Purchase Price Parity [PPP] $416), plus CA$179 (US PPP $144) for travel and CA$84 (US PPP $67) for parking. Patients self-reporting high financial burden had total OOPCs (33%), of CA$961 (US PPP $772), while low-burden participants (66%) had OOPCs of CA$300 (US PPP $241). “Worst burden” respondents spent a mean of 50.7% of their monthly income on OOPCs (median 20.8%). Among the 29.4% who took time off work, patients averaged 18.0 days off. Among the 26.0% of patients whose caregivers took time off work, caregivers averaged 11.5 days off. Lastly, 41% of all patients had to reduce spending. Fifty-two per cent of those who reduced spending were families earning < CA$50,000/year.

**Conclusions:**

In our Canadian sample, high levels of financial burden exist for 33% of patients, and the severity of burden is higher for those with lower household incomes.

**Supplementary Information:**

The online version contains supplementary material available at 10.1007/s00520-020-05907-x.

## Introduction

Cancer treatment consumes significant public and private expenditures, with the most recent published direct medical costs for cancer care in Canada estimated at CA$7.5 billion for 2012 [1]. This does not account for indirect costs related to lost income borne by patients and caregivers, for which the only Canadian estimates are from 2009 and suggest burdens between CA$2.95 and CA$3.18 billion [2].

Access to publicly funded healthcare, as in Canada, implies that financial burden should not limit the delivery of, or access to, cancer treatment. However, several Canadian studies have suggested this is not so [3–6]. Substantial economic burden or out-of-pocket costs (OOPCs) exist in Canada and other publicly funded countries, like Australia, Ireland, and the UK [7–11]. Financial burdens exist for patients with cancer and their families in Canada because not all aspects of care are fully funded. For example, 18–64 years old are not covered for ambulatory drugs in 7 of 10 provinces, homecare has monthly limits in all provinces, and certain diagnostics and procedures are not funded by provincial governments. Co-payments, unfunded care, and lost income for patients and families can result in financial challenges. Hence, policies and programmes such as income replacement and means-based medical care are in place to partially mitigate these financial shocks to patients and their families in most provinces.

The Canada Health Act (CHA) [12] defines terms and conditions which must be met by provincial plans to receive federal funding. The CHA specifies health services (medically required hospital and physician services only) which the federal government agreed to partially fund with the provinces. When the CHA came into effect in 1984, most health services were delivered by physicians or in hospitals. That model is less applicable today, as provision and location of care are evolving. Healthcare services delivered outside the hospital/physician model and the requirements of the CHA include outpatient prescription drugs, homecare, allied healthcare, complementary/alternative medicines (CAM), vitamins/supplements, devices, family support, and other direct treatment-related charges (e.g. private hospital room charges). Gaps in these services exist and have financial consequences for patients and their families (e.g., reductions in monthly limits for covered homecare services, higher deductibles for drugs in private plans).

Gaps and challenges have been examined in Canada [3–6] and elsewhere [7–11], but few studies examined all aspects of financial burden (often labelled financial toxicity in extreme cases) [13]. To develop a comprehensive picture, we included OOPCs, lost income, and treatment-related travel as these were commonly measured in most studies highlighted in a recent systematic review of expenses in developed countries [14].

The intent of this research was to (1) quantify financial burdens in the current Canadian environment; (2) describe patient-reported levels of financial burden; (3) compare differences in actual and perceived financial burden across income, education, and age categories; (4) compare actual and perceived differences across tumour types (breast, colorectal, lung, prostate); and (5) examine the impact of actual and perceived financial burden on patients’ and caregivers’ incomes. Ongoing measurement of these patient costs is important as they have increased in the last few decades as government budgets are constrained and pharmaceuticals and device costs have increased [15]. We included each of these analyses as they represent primary results and factors that are highlighted in literature as important independent variables [10, 16–20].

## Patients and methods

### Patient population

#### Eligibility

Eligible participants were 18 years or older, able to read and write English, with a minimum of 4 weeks of cancer treatment (ideally still on active treatment), and for a diagnosis of breast, colorectal, lung, or prostate cancer (the most common tumours, representing 48% of all cancers in Canada) [21].

#### Recruitment

Eligible participants (*N* = 901) were enrolled at 20 Canadian cancer centres (4 in BC, 6 in Alberta, 1 in Saskatchewan, 1 in Manitoba, 6 in Ontario, 2 in the Atlantic provinces) between May 1, 2016, and May 31, 2019. Centres accrued participants over a 3- to 18-month period; start dates varied based on local ethics board approval. Participants were recruited though various avenues: during cancer clinic visits when providers or research associates (RA) were available; via posters directing patients to an online data entry tool; through mail, using registry data (Manitoba only); or through the use of the Internet panels (Recruited by Asking Canadians; https://www.askingcanadians.com). Cancer clinics were instructed to accrue equal numbers of each tumour type, recognizing this would not be possible in all centres. Variations in available patients, willingness to participate in research, and current health status impacted recruitment. Although the majority of patients were on active treatment (68%), an appeal by physicians and patients who still had expenditures after active treatment resulted in a subset of patients beyond active treatment (32%). With the diverse recruitment strategies, recruitment rates could not be calculated, due to the lack of information about the denominator. Selected local research ethics boards approved a small compensation for participants (i.e., $10 parking vouchers or $10 coffee cards),

### Data sources

The Patient Self-Administered Financial Effects questionnaire (P-SAFE v7.2.4) was used to capture data in-person and online. The availability of both paper and electronic methods allowed patient choice for participation. The online tool met all Canadian requirements for security and privacy regulations, including storage at Canadian data centres. The questionnaire was designed based on previous work done by Birenbaum [22] and Moore [23] and authors’ experience with earlier versions of the P-SAFE [3]. Although other tools are available to measure financial burden, they are less comprehensive, and many of the authors have experience with the P-SAFE tool from past studies. We also have previous data from this tool with which to compare current results.

The P-SAFE v7.2.4 questionnaire (see Electronic Supplementary Material) is a comprehensive measure including 31 questions, some with multiple parts. It includes details on patient demographics (age, gender, education, income, employment status, marital status, living arrangements, residential geography), general health, duration of current cancer treatment, current treatments received (chemotherapy, radiation, surgery, doctor visits, emergency room visits, hospitalizations, in-home nursing services, physiotherapy services), level of insurance coverage, employment details, OOPCs, perceived financial burden, decisions to forego care, and time lost from work for patients and their caregivers. Information on stage of disease was not captured in this self-reported questionnaire, as it was thought that “patient-reported” cancer stage is unreliable. Due to concerns about potential reduced recruitment related to privacy concerns, we did not request access to patient files to determine stage of disease.

OOPCs were classified by “type of expense” into the following categories: prescription drugs, in-home healthcare, homemaking services, complementary and alternative medicine (CAM), vitamins and supplements, family care, other health professionals, accommodations/meals, devices/equipment, and “other” costs. The perceived burden question offered five choices: “no financial difficulty” (none), “small financial difficulty” (small), “somewhat of a financial difficulty” (somewhat), “large financial difficulty” (large), and “worst possible financial difficulty” (worst).

Questions on time lost from work asked participants to calculate reduced days and/or reduced hours in the previous 28 days, and whether government or employer partial or full salary was available for both patients and up to three caregivers. An iterative process (4 cycles) was used for development of concept clarity, explanation text accuracy, and longitudinal congruence (face, content, and predictive validity) for the questionnaire and was assessed as high (*n* = 35) including up to 4 applications/visits per person over 3 months (J Pole et al., publication in progress).

### Calculations and scoring

Some variables required calculations to determine costs. OOPCs were calculated as the sum of drugs, homecare, homemaking, CAM, other health professionals, vitamins/supplements, family care, accommodations, devices, and “other” costs. Imputed travel costs were calculated based on travel distance to the clinic, multiplied by the number of trips, and then multiplied by CA$0.58/Km(Canada Revenue Agency mileage rate at midpoint of recruitment) (https://www.canada.ca/en/revenue-agency/services/tax/businesses/topics/payroll/benefits-allowances/automobile/automobile-motor-vehicle-allowances/automobile-allowance-rates.html). Parking/fares were calculated based on cost per return trip multiplied by the number of trips in the previous 28 days. The calculation of lost income was based on days off work (reported as mean and median lost days). Income loss was calculated as number of days multiplied by the national average daily income rate, based on 235 workdays per year (https://www150.statcan.gc.ca/t1/tbl1/en/tv.action?pid=1110023901). In this regard, the income loss estimates are likely high estimates as some partial compensation would have diminished income losses to families.

The average family income for each participant was estimated by calculating the midpoint of the “family income” category chosen in the questionnaire. The value for those earning > CA$100,000/year was entered as CA$110,000 (a conservative estimate). Income was dichotomized based on scatterplot observations and confirmed by *t* tests at CA$50,000 per year. For “patient perceived burden,” responses were grouped into “high” and “low” burden, based on the percentage of monthly family income (cut-off at median of 3%). This cut-off is frequently used for co-pay adjustments in low-income drug plans (https://www.gov.mb.ca/health/pharmacare/estimator.html). calculation of purchase price parity (PPP) with the USA was obtained from the OECD (https://data.oecd.org/conversion/purchasing-power-parities-ppp.htm).

### Statistical and data analyses

#### Descriptive statistics

Information on participants demographics (age, income categories, education, and gender), tumour type, treatment patterns, and information on the level of burden for participants, including distributions and income effects, is presented as means, medians, standard deviations, and ranges (Tables 1 and 2).

**Table 1:** Study population: demographic characteristics by study sample and tumour type

		Total (*n* = 901)	Breast (*n* = 344)	Colorectal (*n* = 183)	Lung (*n* = 158)	Prostate (*n* = 216)
	Age (SD)	61.3 (14.6)	55.5 (14.2)	59.0 (13.7)	65.3 (13.7)	67.6 (12.7)
	Age range	20–92	20–84	25–92	20–89	23–90
	Male	414	6	113	81	214
	Female	481	336	70	75	0
	Treatment duration (SD)	318 days (328)	307 days (322)	320 days (311)	339 days (349)	318 days (339)
Education		*n* (%)	*n* (%)	*n* (%)	*n* (%)	*n* (%)
	Elementary	19 (2.1)	2 (0.6)	3 (1.6)	8 (5.1)	6 (2.8)
	Some HS	55 (6.1)	10 (2.9)	15 (8.2)	16 (10.1)	14 (6.5)
	Compl HS	169 (18.8)	68 (19.8)	39 (21.3)	32 (20.2)	30 (13.9)
	Some Univ	170 (18.9)	58 (16.9)	30 (16.4)	38 (24.1)	44 (20.4)
	Compl Univ	367 (40.8)	161 (46.9)	75 (40.1)	52 (32.9)	79 (36.6)
	Post Grad	120 (13.3)	44 (12.8)	21 (11.5)	12 (7.6)	43 (19.9)
Income		*n* (%)	*n* (%)	*n* (%)	*n* (%)	*n* (%)
	$0–19.9 K	65 (7.2)	19 (5.5)	17 (9.3)	21 (13.3)	8 (3.7)
	$20–39.9 K	137 (15.2)	50 (14.6)	33 (18.0)	29 (18.4)	25 (11.6)
	$40–59.9 K	142 (15.8)	54 (15.7)	27 (14.8)	28 (17.7)	33 (15.3)
	$60–79.9 K	134 (14.9)	52 (15.2)	28 (15.3)	24 (13.0)	30 (13.9)
	$80–99.9 K	131 (14.6)	52 (15.2)	22 (12.0)	13 (8.2)	44 (20.4)
	$100 K plus	218 (24.2)	90 (26.2)	42 (23.0)	25 (15.8)	61 (28.2)
	Missing/DK	73 (8.1)	26 (7.6)	14 (7.7)	18 (11.4)	15 (6.9)

*SD* standard deviation; *Some HS* some high school

*Compl HS* completed high school; *Some Univ* some university

*Compl Univ* completed university; *DK* do not know; 5 patients declared gender as “other”

**Table 2:** Total 28-day out-of-pocket cost (excluding travel and parking) and per cent of monthly family income by “burden category”

	*N* [% sample]	Median % of income	$Mean [% income]	Std. Dev.	$Range [% income range]
“None”	394 [44%]	(0.5%)	$214 (4.1%)	$768 (44%)	$0–10,070 (0–238%)
“Small”	209 [23%]	(3%)	$462 (11.1%)	$1202 (32.3%)	$0–12,800 (0–336%)
“Somewhat”	186 [21%]	(9.7%)	$916 (24.4%)	$1812 (50.3%)	$0–13,240 (0–360%)
“Large”	90 [10%]	(14.2%)	$1096 (50.7%)	$2803 (127.3%)	$0–25,240 (0–962%)
“Worst possible”	22 [2%]	(20.8%)	$784 (50.7%)	$1544 (117.7%)	$0–7130 (0–523%)
Total	901 [100%]	(2.6%)	$518 (15.7%)	$1486 (55%)	$0–25,240 (0–962%)
Low vs high burden	*N*	Median	Mean	Std. Dev	Range
Low (none + small)	603	$60	$300	$948	$0–12,800
High (somewhat + large + worst)	298	$360	$961	$2139	$0–25,240

*None* no financial burden; *small* small financial burden; *somewhat* somewhat of a financial burden; *large* large financial burden; *worst possible* worst possible financial burden

#### Analyses of variance (ANOVA)

ANOVAs were performed to identify differences in dependent variables between different groups (independent variables) including tumour type, education, and income. Appropriate statistical tests were applied based on equal or unequal ANOVA variances. Where ANOVA showed statistically significant differences, the Tukey honest statistical difference (HSD) post-hoc test [24] was used. Where *t* tests were undertaken, a variance ratio test was used to determine if variances were equal, and the appropriate *t* test was then applied.

#### Multiple regressions

To improve understanding of the relative influence of multiple independent variables to assist in identifying patients at financial risk, a single multiple linear regression model was structured that included the OOPC as the dependent variable and age, income, and burden as the independent variables.

All analyses were performed using statistical software RStudio v1.2.1335 [2019] (based on R platform v3.6).

### Research ethics

Approval was obtained from McMaster University’s Hamilton Integrated Research Ethics Board (HiREB #1743). Sites local research ethics approvals were obtained from each cancer centre.

## Results

### Participants

A total of 901 individuals participated in the survey. Participants chose not to answer some questions leaving some data fields incomplete (e.g., 8.2% selected “Do not know/missing” for income). Data available through the hosting website revealed the questionnaire took 20–35 min to complete online.

Participants were evenly divided between male (45.6%) and female (53.8%) (5 patients declared gender “other”). The male/female mix varies by tumour type (Table 1).Mean age was 61.3 years with variability between tumour types. Those with breast cancer are youngest (55.5 years; SD 14.2), and those with prostate cancer are oldest (67.6 years; SD 12.7) [Table 1]. ANOVA results reveal a statistically significant age difference between tumour types (*F*(3,427) = 41.95, *p* < 0.001). Participants had a skewed education distribution, with 8.2% of the sample having less than a high school education and 73.0% having at least some university/college exposure (Table 1).

The average duration of treatment for the participants was just under 1 year (318 days; SD 328). The range of treatment duration was 25 days to 2.7 years.

### National participant expenditures

Aggregate mean monthly OOPCs for all patients with cancer are CA$518/28 days (SD 1486) [US PPP $416] with an additional CA$179 (SD 737) [US PPP $144] related to imputed travel, CA$84 (SD 266) [US PPP $67] for parking/fares, and a combined CA$1733 for patient and caregiver lost income (Table 2; salaries not converted to USD). The resulting overall cost/28 days was CA$2514. Although those in early treatment (< 1 year) had slightly higher costs than those beyond a year, the difference was not statistically significant (CA$574 vs. CA$404; *t*(510) = 1.53, *p* = 0.1269).

### Dichotomizing burden

An analysis of burden across family income was undertaken where low-burden responses (“none” and “small”) were compared to higher burden responses (“somewhat”, “large”, and “worst possible”). This was done because those reporting a “somewhat”, “large”, or “worst possible” burden had OOPC of a relatively high median percentage of family income (9.7% (somewhat), 14.2% (large), and 20.8% (worst possible)). Conversely, those reporting lower burden had a median OOPC of less than 3% of their family income (0.5% (none) and 3% (small)). Therefore, grouping them in this way seemed justified. This aggregated analysis shows a statistically significant difference (*F*(2,251) = 5.29 *p* = 0.0056) between low and high-burden groups in the percentage of monthly income spent on OOPC related to their cancer (Table 2).

### Perceived participant financial burden

More than 33% of participants perceive their financial burden to be high (Table 3). Those with lowest burden (among “no burden”) had mean OOPCs of CA$214, and those with highest (“large burden”) had mean OOPCs of CA$1096 [Table 2]. The difference between total OOPCs between groups by self-reported financial burden was statistically significant (*F*(4,121) = 9.68, *p* < 0.001), with differences in total OOPC between “small” and “large” (*p* = 0.005); “none” and “large” (*p* < 0.001); “none” and “somewhat” (*p* < 0.001); and “somewhat” and “small” burdens (*p* = 0.017). Mean OOPCs are higher for participants who perceived a “somewhat, large, or worst possible” burden than for others (Table 3). Participants who responded that the burden was “somewhat, large, or worst possible” reported a mean OOPC of CA$961/28 day (SD 2139) [US PPP $772], while those describing “none” or “small” burden spent CA$300 (SD 948) [US PPP $241] as outlined in Table 2. ANOVA comparing individual cost categories for OOPCs by self-reported burden were statistically significant for “prescription drugs” (*F*(4,67) = 3.45 *p* = 0.013), “vitamins/supplements” (*F*(4,65) = 7.49, *p* < 0.001), and “CAM” (*F*(4,37) = 371 *p* = 0.012) where those with high burden spent more, with no significant differences for the other cost categories.

**Table 3:** Patients’ perceived level of burden, by income category

	None/Small (*n*)	Somewhat/Large/Worst (*n*)
$0–$19.9 K	43.1% (28)	56.9% (37)
$20–$39.9 K	53.3% (73)	46.7% (64)
$40–$59.9 K	66.2% (94)	33.8% (48)
$60–$79.9 K	64.9% (87)	32.1% (47)
$80–$99.9 K	72.5% (95)	27.5% (36)
$100 K and over	80.3% (175)	19.7% (43)
Do not know/missing	68.5% (50)	31.5% (23)
Total*	66% (602)	33% (298)

*1 non-response

Analysis of the percentage of monthly family income by burden category is undertaken (Table 3). Only actual incurred costs were used in this analysis; imputed travel costs were omitted as they did not necessarily represent OOPCs during that month. This analysis showed a clear relationship between percentage of monthly income spent and perceived burden. Participants with “no burden” spent a mean 4.1% (median 0.5%) of their monthly income, and those with a “worst possible” burden spent a mean 50.7% (median 20.8%) of their monthly income on OOPC.

### Independent variables: Income, education, and age effects

ANOVAs on OOPCs revealed statistically significant differences with income (*F*(11,138) = 4.92, *p* < 0.001) and education (*F*(5,206) = 6.42; *p* < 0.001). When income was dichotomized (under $CA50,000/year vs. over), those with lower income spent less out-of-pocket (*F*(2,251) = 5.29, *p* = 0.0056). While 56.0% of participants with incomes below CA$20,000 identified the burden as “somewhat, large, or worst possible”, only 22.6% of those with family incomes over CA$80,000 did so (Table 4).

**Table 4:** Mean lost time from work for patients and caregivers by burden and burden category (low vs high)

Burden (*n*; %)	None (394; 44)	Small (209; 23)	Somewhat (186; 21)	Large (90; 10)	Worst (22; 2)
Mean days lost work patient [SD]	3.4 [8.1]	5.3 [9.4]	6.5 [10.1]	9.8 [11.6]	5.6 [10.1]
Mean days lost caregivers [SD]	1.2 [5.3]	2.7 [7.0]	4.7 [11.4]	6.3 [13.6]	6.8 [12.3]
Patient mean days [SD]	4.1 [8.6]		7.4 [10.6]		
Caregiver mean days [SD]	1.7 [6.0]		5.33 [12.2]		

*Low burden* none or small burden; *high burden* somewhat, large, and worst possible burden

When looking at participants’ perceived burden by age, those < 65 years were twice as likely to report the burden as “somewhat, large, or worst possible” (age < 65: 43%; age > 65: 21%).

Participants < 65 years (55%) took considerably more time off work (average 8.21 days) compared to those > 65 years (45%) who took an average of 1.49 days off work (*t*(771) = 12.0; *p* < 0.001). Similarly, caregivers supporting those < 65 took 4.33 days off work, while those supporting patients > 65 took 1.20 days off work (*t*(741) = 5.81; *p* < 0.001). Overall, 26.2% of participants required caregivers to take time off from paid work, rising to 35.2% for participants < under 65 years.

### Differences by tumour type

An ANOVA for total OOPCs across tumour types was not statistically significant (*F*(3,409) = 0.0546; *p* = 0.9832).

### Time lost from work

For participants who worked during the previous 28 days, mean lost time from work was 18.0 days. Moreover, many caregivers also lost time from work; the 26% that took time off averaged 11.5 days off (average 3.0 days off for the entire sample). The 33% of participants who perceived a high cost burden had a significantly higher average number of days off work than the participants in the low-burden category (*t*(494) = 4.77; *p* < 0.001, 7.44 days vs 4.06 days; Table 4). This difference regarding more time off for those with high burden is also seen for caregivers (*t*(369) = 4.84, *p* < 0.001, 5.33 days vs. 1.72 days; Table 4). Income was impacted for those < 65 who were earning more compared to those > 65 (Pearson Chi-square test; *X*^2^(3, *N* = 827) = 7.933, *p* = 0.047). A crude estimate (based on provincial salary averages) of lost income revealed that participants and their families lost on average CA$209 per day of work missed. Using this figure, 26% of participants had family members lose more than CA$2402 in income over 28 days. Additionally, the 29% of participants who were working lost an average of CA$3759 in income over 28 days.

### Multiple regression

Multiple regression analysis was run including age, burden, and income given their potential impact on total OOPCs. Analysis revealed that each has an impact on the amount spent. Participants earning less than CA$40,000, those with a “low” burden, and those > 65 spent less, although some of the observations do not reach statistical significance (Table 5).

**Table 5:** Multiple regression model out-of-pocket costs by age category (under 65 vs. 65 and over), income, and burden category (high vs. low)

	Dependent variable:		Out-of-pocket costs (OOPC)	
Variable	Coefficient (SE)	Beta	95% confidence interval	*P* value
Age > 65 years	-$139.86 (108.93)	1.28	-$30.93, $248.79	*P* > 0.1 NS
Income				
< $20,000	-$517.35 (215.69)	2.40	-$301.66, $733.04	*P* < 0.05
$20,000–$39,999	-$380.48 (166.58)	2.28	-$213.90, $547.07	*P* < 0.05
$40,000–$59,999	-$133.25 (162.84)	0.82	-$296.08, $29.57	*P* > 0.1 NS
$60,000–$79,999	-$264.73 (164.73)	1.61	-$429.45, $100.00	*P* > 0.1 NS
$80,000–$99,999	$6.54 (165.21)	0.04	-$158.68, $171.75	*P* > 0.1 NS
High burden	$766.56 (117.40)	6.53	$649.16, $883.96	*P* < 0.01
Constant	$510.53 (113.37)	4.50	$397.16, $623.90	*P* < 0.01

Observations 827

*R*^2^ 0.064

Adjusted *R*^2^ 0.056

Residual Std. error 1490.401 (df = 819)

F Statistic 7.978 (df = 7; 819) p < 0.01

“$100,000+” is income reference, “small burden” is burden reference, “Under 65” is age reference. *Hypotheses*: Lower OOPC patient 65 years and over; higher income patients higher OOPC; higher burden patients higher OOPC

Standardized beta = coefficient/SE; 74 pts. income was “do not know” (*n* = 73) or “missing” (*n* = 1)

## Discussion

This study sought to identify patient and family financial burdens related to cancer treatment and how they might differ by patient perceived burden, tumour, age, income, and education. Results suggest about one-third of participants find the burden of OOPCs to be a “somewhat, large, or worst possible” burden, despite having publicly funded healthcare; this group reported spending an average of 34% of their monthly income on cancer related costs. Direct and indirect cost elements likely both play a role in patients’ perceived burden [25–27]. The results confirm expenditure as a percentage of income is greatest for those with low incomes, consistent with the previous research [18]. It is worth noting that most government mean tests for healthcare services in Canada use 3–4% of family income as a co-pay cut-off, with values above this qualifying for full coverage [28]. Finally, the limited literature that does exist on patients with cancers’ OOPC is mostly in a predominately private, for-profit healthcare setting, so this research adds significantly to our understanding of the financial impact when the majority of care is publicly funded.

Although previous work suggested cost differences exist across tumour types [3], in our study, no significant difference exists between cancer types. It could be speculated that as costs have increased for people across all tumour types, differences in costs between tumour groups have diminished. This could be partially influenced by increases in both prescription drug expenditures and homecare expenditures, but further studies are required to confirm this. It is also possible that differences in stage of disease, types of treatments, and duration of illness may be confounders. Furthermore, the regression using age, income, and burden showed that OOPCs are influenced by both income and burden, but age was not significant. These results are consistent with other literature regarding income and burden effects [4].

The majority of patients with cancer did not report any paid employment during the 28-day study period. While for some (particularly seniors), this may reflect they were not in paid employment prior to illness; others experienced a 100% loss of employment income. For those caregivers who worked over the previous 28 days, income losses were on average less than 100%; nevertheless, data suggest caregivers may lose approximately 50% of their potential workdays in any given month to assist in patient care (26% of our sample). The caregivers’ percentage increases to 35% when the patient is < 65 years.

Patient care needed at home is likely a cause of this lost time from work for caregivers. In Ontario, this is an 85% (67% inflation adjusted) increase from the numbers seen in an earlier study [3] with the same four cancer groups. This issue is likely more severe in cases where the cancer more closely resembles a chronic condition, resulting in longer term potential for out-of-pocket payments, family members assuming caregiver roles, or decisions to forego caregiving support. The average duration of treatment for patients in this study was just less than 1 year (with a range from 25 days to 2.7 years), a time period that exceeds coverage for most current publicly funded homecare programmes and income replacement programmes. Caregiver income replacement programmes supporting adults at “end of life” cover only 15 to 26 weeks, (https://www.canada.ca/en/services/benefits/ei/caregiving.html) leaving many caregivers with incomplete coverage or resorting to someone unqualified. In cases where family members provided support, public homecare may not have been provided or was not sufficient, patients may not have qualified, or patients/families did not know how to access such care. Of note, the number of hours of supportive services paid by the Ontario government has decreased since 2003, resulting in patients either paying privately for support or using informal caregivers (family and friends). Preferences and cultural differences may also play a role in this change, although this cannot be stated with confidence, as this study did not measure these factors. This raises important issues about OOPCs and income losses particularly for those patients with prolonged illnesses.

### Limitations and future research

This sample was taken from twenty cancer clinics and included the four most common tumour types in Canada. Patients treated outside cancer clinics or patients with other cancers were not included. We do not know if their costs would be higher or lower or in the same categories. Lack of information on those who did not participate is also problematic, but as resources were scarce and privacy laws make these requests challenging, we may have an unmeasured bias in our sampling. Also, urban cancer centres enrolled more than rural sites, although many urban centres treat rural dwelling patients who face the potential for added transportation costs [14]. There are other factors that may impact patients’ disposable income including loans, education savings, and personal savings. This information was captured; however, we only captured the use of these resources, not the monetary value.

Most cancer treatments that require aggressive chemotherapy would, by definition, require a significant expense related to prescription drugs (especially for oral anti-emetics and oral chemotherapy agents). Current examples of this with newer treatments have highlighted longer treatment times and higher costs [15]. In this regard, it is expected that gaps identified in this research would also be found in other tumour types. Demographics can also play a role, as some cancers occur in younger populations who are more likely to have lower incomes, limited savings, and be uninsured or underinsured. Lastly, this study did not capture information on race or ethnicity. It is advised that future work in this area should include these details, where possible.

Conversion from Canadian to US dollars is somewhat problematic as exchange rates do not accurately reflect buying power. We used purchase price parity (PPP) tables to convert. This approach has limitations, as PPP values are based on a basket of goods despite the variability within that basket. Hence, PPP conversions for healthcare could be an overestimate or an underestimate of actual buying power.

Information on stage of disease was not captured, although this study did capture a proxy for stage of disease. However, until we can match this proxy to disease stage (ongoing evaluation in a subset of patients), commenting on this aspect of the data is not possible. Literature has shown that costs associated with care for lung, colorectal, breast, prostate, and bladder cancer patients tend to be highest in the first 6 months following diagnosis and in the last 12 months before death [29]. Patients were recruited in this study throughout the life cycle of their illness; hence, significant variation in healthcare resource use should be expected, which may impact their OOPC as well as their perceived financial burden. Some costs were episodic and were captured early in a patients’ treatment, while others were captured later in their treatment. These variations make it more difficult to elucidate factors that determine those patients most at risk for significant financial burdens.

Due to privacy issues, sites were not able to capture those who declined participation. Therefore, there is a risk of bias in this sample, and to some degree, this has been seen in an over representation of those in the higher income brackets (Table 1). Although the intention was to recruit patients equally from all four tumour groups, this was not attained due to limited availability or poor health status of patients in some centres. Recruitment strategies also differed with some centres recruiting the majority of patients using paper copies, while others used online enrollment. Although no statistical difference across recruitment methods was seen, nonetheless, this may have introduced bias. Additionally, given the use of *p* < 0.05 has been challenged recently [30], some results just over this threshold and declared not significant could be interpreted differently.

Lastly, consistent with earlier studies [3, 4], income loss calculations are based on actual time lost from work. This income loss estimate is likely overstated, and calculations adjusting for government and private payor coverage would be more accurate. However, each of these calculations requires a series of assumptions and models which will be addressed in a subsequent publication.

### Policy implications

In Canada, some programmes are designed to assist patients with high financial health-related burdens. They include special means tested drug funding programmes, age-related programmes, healthcare funding programmes for patients with work-related illnesses, and special healthcare funding for persons who are out of work (http://www.health.gov.on.ca/en/pro/programs/drugs/funded_drug/funded_drug.aspx, Accessed July 28, 2020). Whether participants with high financial burden were eligible for and/or aware of such assistance is worthy of further investigation. However, previous work has suggested that about three-quarters of patients are not aware of aid programmes [31].

The high financial burden reported by one-third of patients is likely multi-factorial and influenced by the limited availability of supportive services and higher prices for pharmaceuticals. Psychosocial distress, which has been shown to be as high as 43% in lung cancer patients [32], is an additional factor that may impact perceived burden. There may be a valuable opportunity to minimize perceived patient burden through more extensive supportive care programmes and better pharmaceutical coverage.

Loss of caregivers’ income while delivering necessary services at home contributes greatly to perceived burden. In December 2017, the Federal Minister of Health announced a 5-year CA$691 million grant to cover supportive leave for family delivering end-of-life care for up to 15 weeks for critically ill patients and up to 26 weeks for end-of-life support (https://www.newswire.ca/news-releases/more-choice-and-flexibility-for-families-and-caregivers-starting-december-3-2017-656401883.html, accessed July 28, 2020). This will address needs of many patients with advanced disease. Even so, maximum payments are set at 55% of full income, which may still result in significant financial burden for patients and their families. Moreover, most common cancers have treatment cycles of 6–12 months and follow-up treatment often for years, well beyond the limits of these new federal programmes.

These study results suggest the boundaries of healthcare can spill over into, or are influenced by, other social programmes, such as those associated with income replacement. It raises the question of whether health policy makers should consider the influence of programmes outside the Ministry of Health silo when evaluating the comprehensiveness of publicly funded healthcare for illnesses like cancer.

This research answers a number of questions about the size and frequency of financial burdens in Canada, generating opportunities for future research. These findings are consistent with other developed countries in terms of income losses, yet they differ in terms of OOPCs, with Canada being more expensive than countries like Ireland [10] or the UK [11] but less expensive than the USA [13]. Key findings should provide useful information to policy makers and allow a closer evaluation of existing programmes in light of the success or failure of programmes to mitigate patient cancer-related costs across the country.

## Supplementary Information

ESM 1 (DOCX 72 kb)

## Data Availability

The dataset generated during the current study are available from the corresponding author on reasonable request.

## References

[1] de Oliveira C, Weir S, Rangrej J, Krahn MD, Mittmann N, Hoch JS, Chan KKW, Peacock S (2018). The economic burden of cancer care in Canada: a population-based cost study. CMAJ Open.

[2] Hopkins RB, Goeree R, Longo CJ (2010). Estimating the national wage loss from cancer in Canada. Curr Oncol.

[3] Longo CJ, Fitch M, Deber RB, Williams AP (2006). Financial and family burden associated with cancer treatment in Ontario, Canada. Support Care Cancer.

[4] Longo CJ, Deber R, Fitch M (2007). An examination of cancer patients’ monthly “out-of-pocket” costs in Ontario, Canada. J Cancer Care.

[5] Lauzier S, Levesque P, Drolet M, Coyle D, Brisson J, Mâsse B, Robidoux A, Robert J, Maunsell E (2011). Out-of-pocket costs for accessing adjuvant radiotherapy among Canadian women with breast cancer. J Clin Oncol.

[6] Butler L, Downe-Wamboldt B, Melanson P, Coulter L, Keefe J, Singleton J, Bell D (2006). Prevalence, correlates, and costs of patients with poor adjustment to mixed cancers. Cancer Nurs.

[7] Gordon L, Scuffham P, Hayes S, Newman B (2007). Exploring the economic impact of breast cancers during the 18 months following diagnosis. Psycho-Oncology.

[8] Gordon LG, Ferguson M, Chambers SK, Dunn J (2009). Fuel, beds, meals and meds: out-of-pocket expenses for patients with cancer in rural Queensland. Cancer Forum.

[9] Hanly P, Ceilleachair AO, Skally M (2013). How much does it cost to care for survivors of colorectal cancer? Caregiver’s time, travel and out-of-pocket costs. Support Care Cancer.

[10] O’Ceilleachair A, Hanly P, Skally M (2017). Counting the cost of cancer: out-of-pocket payments made by colorectal cancer survivors. Support Care Cancer.

[11] Marti J, Hall PS, Hamilton P, Hulme CT, Jones H, Velikova G, Ashley L, Wright P (2016). The economic burden of cancer in the UK: a study of survivors treated with curative intent. Psychooncology.

[12] Government of Canada. (1984). Canada Health Act. Retrieved from http://www.hc-sc.gc.ca/medicare/home.htm. Accessed 10 Aug 2020

[13] Zafar SY, Abernethy AP (2013). Financial toxicity, part I: a new name for a growing problem. Oncology (Williston Park).

[14] Longo CJ, Fitch MI, Banfield L, Hanly P, Yabroff KR, Sharp L (2020). Financial toxicity associated with a cancer diagnosis in publicly funded healthcare countries: a systematic review. Support Care Cancer.

[15] Tran G, Zafar SY (2018) Financial toxicity and implications for cancer care in the era of molecular and immune therapies. Ann Transl Med 6(9). 10.21037/atm.2018.03.28 10.21037/atm.2018.03.28 PMC5985271 29911114

[16] Newton JC, Johnson CE, Hohnen H, Bulsara M, Ives A, McKiernan S, Platt V, McConigley R, Slavova-Azmanova NS, Saunders C (2018). Out-of-pocket expenses experienced by rural Western Australians diagnosed with cancer. Support Care Cancer.

[17] Park J-H, Park E-C, Park J-H, Kim SG, Lee SY (2008). Job loss and re-employment of cancer patients in Korean employees: a nationwide retrospective cohort study. J Clin Oncol.

[18] de Oliveira C, Bremner KE, Ni A, Alibhai SMH, Laporte A, Krahn MD (2014). Patient time and out-of-pocket costs for long-term prostate cancer survivors in Ontario, Canada. J Cancer Surviv.

[19] Barbaret C, Brosse C, Rhondali W, Ruer M, Monsarrat L, Michaud P, Schott AM, Delgado-Guay M, Bruera E, Sanchez S, Filbet M (2017). Financial distress in patients with advanced cancer. PLoS One [Electronic Resource].

[20] Arndt V, Koch-Gallenkamp L, Bertram H, et al (2019) Return to work after cancer. A multi-regional population-based study from Germany 10.1080/0284186X.2018.1557341 30777496

[21] Cancer statistics at a glance (https://www.cancer.ca/en/cancer-information/cancer-101/cancer-statistics-at-a-glance/?region=on). Accessed 10 Aug 2020

[22] Birenbaum LK (1992). Terminal care costs in childhood cancer. Pediatr Nurs.

[23] Moore KA (1999). Breast cancer patients’ out-of-pocket expenses. Cancer Nurs.

[24] Tukey JW (1984) The collected works of John W. Tukey. Taylor & Francis

[25] Guidry JJ, Aday LA, Zhang D, Winn RJ (1998). Cost considerations as potential barriers to cancer treatment. Cancer Pract.

[26] Houts PS, Lipton A, Harvey HA, Martin B, Simmonds MA, Dixon RH, Longo S, Andrews T, Gordon RA, Meloy J, Hoffman SL (1984). Non-medical costs to patients and their families associated with outpatient chemotherapy. Cancer.

[27] Stommel M, Given CW, Given BA (1993). The cost of cancer home care to families. Cancer.

[28] Law MR, Cheng L, Worthington H, Mamdani M, McGrail KM, Chan FKI, Majumdar SR (2017). Impact of income-based deductibles on drug use and health care utilization among older adults. CMAJ.

[29] Riley GF, Potosky AL, Lubitz JD, Kessler LG (1995). Medicare payments from diagnosis to death for aged cancer patients by state at diagnosis. Med Care.

[30] Harrison AJ, McErlain-Naylor SA, Bradshaw EJ (2020). Recommendations for statistical analysis involving null hypothesis significance testing. Sports Biomech.

[31] Housser EM, Mathews M, LeMessurier J, Young S, Hawboldt J, West R (2013). Responses by breast and prostate cancer patients to out-of-pocket costs in Newfoundland and Labrador. Curr Oncol.

[32] Carlson LE, Bultz BD (2003). Cancer distress screening: needs, models and methods. J Psychosom Res.

