# Correction to: Patient and family financial burden associated with cancer treatment in Canada: a national study

**DOI:** 10.1007/s00520-021-06031-0

**Published:** 2021-02-15

**Authors:** Christopher J. Longo, Margaret I. Fitch, Jonathan M. Loree, Linda E. Carlson, Donna Turner, Winson Y. Cheung, Darin Gopaul, Janet Ellis, Jolie Ringash, Maria Mathews, Jim Wright, Christiaan Stevens, David D’Souza, Robin Urquhart, Tuhin Maity, Fanor Balderrama, Evette Haddad

**Affiliations:** 1 DeGroote School of Business–Health Policy & Management, McMaster University, 4350 South Service Rd, Burlington, Ontario L7L 5R8 Canada; 2 Dalla Lana School of Public Health, University of Toronto, Toronto, Canada; 3 Bloomberg Faculty of Nursing, University of Toronto, Toronto, Ontario M4C 4V9 Canada; 4 Department of Medicine, Division of Medical Oncology, BC Cancer/University of British Columbia, 600 West 10th Avenue, Vancouver, British Columbia V5Z4E6 Canada; 5 Department of Oncology, Cummings School of Medicine, University of Calgary, 2202 2nd St SW, Calgary, Alberta T2S 3C1 Canada; 6 Department of Community Health Sciences, Rady Faculty of Health Sciences, University of Manitoba, 675 McDermot Avenue, Winnipeg, MB R3E 0V9 Canada; 7 Department of Oncology, University of Calgary, 1331–29 Street NW, Calgary, Alberta T2N 4N2 Canada; 8 Grand River Regional Cancer Centre, Kitchener, Ontario N2G 1G3 Canada; 9 Odette Cancer Centre, Sunnybrook Health Sciences Centre, Toronto, Ontario Canada; 10 Princess Margaret Cancer Centre/UHN, 610 University Ave, Toronto, Ontario M5G 2M9 Canada; 11 Department of Family Medicine, Western University, London, Ontario Canada; 12 Juravinski Cancer Centre, McMaster University, Hamilton, Canada; 13 Department of Radiation Oncology, University of Toronto, Barrie, Ontario L4M 6M2 Canada; 14 London Health Sciences Centre, Western University, London, Ontario Canada; 15 Department of Surgery, Dalhousie University, Room 8-032, Centennial Building, 1276 South Park St., Halifax, Nova Scotia B3H 2Y9 Canada; 16 Department of Health Research Methods, Evidence, and Impact, McMaster University, 1280 Main Street West, Hamilton, Ontario L8S 4L8 Canada

**Correction to: Supportive Care in Cancer**

10.1007/s00520-020-05907-x

Table 5 in the original missed “minus” signs in line 1 (age), line 2-6 (income).

**Table 5:** Multiple regression model out-of-pocket costs by age category (under 65 vs. 65 and over), income, and burden category (High vs. Low)

	**Dependent variable:**		**Out-of-pocket costs (OOPC)**	
Variable	Coefficient (SE)	Beta	95% Confidence Interval	P value
Age >65 years	-$139.86 (108.93)	1.28	-$30.93, -$248.79	P>0.1 NS
**Income**				
<$20,000	-$517.35 (215.69)	2.40	-$301.66, -$733.04	P<0.05
$20,000-$39,999	-$380.48 (166.58)	2.28	-$213.90, -$547.07	P<0.05
$40,000-$59,999	-$133.25 (162.84)	0.82	-$296.08, $29.57	P>0.1 NS
$60,000-$79,999	-$264.73 (164.73)	1.61	-$429.45, -$100.00	P>0.1 NS
$80,000-$99,999	$6.54 (165.21)	0.04	-$158.68, $171.75	P>0.1 NS
High Burden	$766.56 (117.40)	6.53	$649.16, $883.96	P<0.01
Constant	$510.53 (113.37)	4.50	$397.16, $623.90	P<0.01

Observations 827

R2 0.064

Adjusted R2 0.056

Residual Std. Error 1,490.401 (df = 819)

F Statistic 7.978 (df = 7; 819) p<0.01

“$100,000+” is income reference, “small burden” is burden reference, “Under 65” is age reference. *Hypotheses*: Lower OOPC patient 65 years and over; higher income patients higher OOPC; higher burden patients higher OOPC.

NOTES: Standardized beta = coefficient/SE; 74 pts income was “don’t know” (n=73) or “missing” (n=1)

The correct table is shown below:

The original article has been corrected.

